# Definitive chemoradiotherapy for squamous cell carcinoma of the esophagus: outcomes for borderline-resectable disease

**DOI:** 10.1093/jrr/rraa008

**Published:** 2020-04-06

**Authors:** Satoshi Ishikura, Takuhito Kondo, Taro Murai, Yoshiyuki Ozawa, Takeshi Yanagi, Chikao Sugie, Akifumi Miyakawa, Yuta Shibamoto

**Affiliations:** 1 Department of Radiology, Nagoya City University Graduate School of Medical Sciences, Nagoya, Aichi 467-8601, Japan; 2 Department of Proton, Narita Memorial Proton Center, Toyohashi, Aichi 441-8021, Japan; 3 Department of Radiology, Nagoya Daini Red Cross Hospital, Nagoya, Aichi 466-8650, Japan; 4 Department of Radiation Oncology, National Hospital Organization Nagoya Medical Center, Nagoya, Aichi 460-0001, Japan

**Keywords:** cancer of esophagus, chemoradiotherapy, induction chemotherapy, surgery, organ-sparing treatments

## Abstract

Definitive chemoradiotherapy (dCRT) is the standard treatment for unresectable esophageal cancer. Induction chemotherapy has been actively investigated for borderline-resectable and unresectable disease, but the superiority over dCRT has yet to be confirmed. The purpose of this study was to evaluate the outcome of dCRT with special interest in borderline-resectable disease. Patients with esophageal cancer treated with dCRT between January 2004 and November 2016 were included in this retrospective analysis. Chemotherapy consisted of two cycles of cisplatin (70–75 mg/m^2^) on day 1 and 5-fluorouracil (700–1000 mg/m^2^ per day) on days 1–4 or low-dose cisplatin (10 mg/m^2^ per day) and 5-fluorouracil (175 mg/m^2^ per day) for 20 days. Radiotherapy was given with a daily fraction of 1.8–2 Gy to a total dose of 50–70 Gy. A total of 104 patients were included: 34 were resectable, 35 were borderline-resectable and 35 were unresectable. Complete response was achieved in 44 patients (42%). Eighteen patients (17%) suffered Grade 2 or greater cardiopulmonary toxicity and seven patients (7%) suffered Grade 3 cardiopulmonary toxicity. At the time of this analysis, 59 patients were dead and 45 were censored. The 3-year overall survival proportions for resectable, borderline-resectable and unresectable patients were 64%, 46% and 21%, respectively. The overall survival for borderline-resectable patients with complete response and noncomplete response was significantly different (*P* < 0.001), with 3-year survival of 70% and 8%, respectively. The overall survival for complete response patients with borderline-resectable disease was encouraging. Further investigation to find a subgroup fit for esophagus-preserving treatment is warranted.

## INTRODUCTION

Definitive chemoradiotherapy (dCRT) is the standard treatment for nonmetastatic squamous cell carcinoma of the esophagus when the disease is unresectable or patients prefer nonsurgical treatment [1–5]. Phase III studies to evaluate the non-inferiority of dCRT compared with chemoradiotherapy (CRT) followed by surgery are ongoing for resectable disease. The treatment outcome for locally advanced disease is still dismal, and induction chemotherapy with or without concurrent radiotherapy has been investigated for unresectable disease expecting conversion surgery [6–10]; however, the superiority of this approach over dCRT is yet to be confirmed. An issue that particularly interests us is the decision of resectability. In pretreatment evaluation, computed tomography (CT) of the neck, chest and abdomen is commonly used to determine the clinical stage and resectability of the tumor, but sometimes invasion to the surrounding normal tissues, such as the descending aorta and/or bronchial tree, is suspected but not definite, which causes large inter-observer variations among diagnostic radiologists and surgeons and leads to uncertainty in the decision of resectability [11–13]. These cases are expected to show better outcome compared with definite unresectable disease and worse outcome compared with definite resectable disease. Thus, the concept of borderline-resectable disease was proposed, with a treatment strategy that is different from resectable and unresectable disease being sought. The number of reports focusing on borderline-resectable disease is limited, and more data are needed to establish the standard treatment, including an esophagus-preserving strategy. The purpose of this study was to evaluate the outcome of dCRT, with special interest in borderline-resectable disease, and to generate a hypothesis that overall survival after dCRT is not inferior to that after surgery following induction therapy in patients with borderline-resectable disease who responded well to CRT.

## MATERIALS AND METHODS

### Patient population and pretreatment evaluation

Patients with newly diagnosed squamous cell carcinoma of the esophagus and treated with dCRT between January 2004 and November 2016 at our institution were extracted from our database and included in this retrospective analysis. Pretreatment evaluation included endoscopy of the esophagus and CT of the neck, chest and abdomen. Bronchoscopy, magnetic resonance imaging (MRI) of the esophagus and ^18^F-fluorodeoxyglucose positron emission tomography with CT (PET-CT) of the whole body were optional. The seventh edition of the Union for International Cancer Control (UICC)-Tumor, Node, Metastases (TNM) staging system was used.

### Treatment

Concurrent chemotherapy with radiotherapy consisted of two cycles of cisplatin (70 mg/m^2^) on day 1 and 5-fluorouracil (5-FU) (700 mg/m^2^ per day) on days 1–4 administered every 4 weeks; two cycles of cisplatin (75 mg/m^2^) on day 1 and 5-FU (1000 mg/m^2^ per day) on days 1–4 every 4 weeks; or low-dose cisplatin (10 mg/m^2^ per day) and 5-FU (175 mg/m^2^ per day), 5 days a week for 20 days. Low-dose cisplatin/5-FU was used in the earlier period, and the regimen was selected at the discretion of the treating physicians based on the disease stage and medical condition of the patient. Two additional cycles of maintenance chemotherapy were performed in selected patients. Radiotherapy was given with conventional techniques, such as anterior–posterior opposed irradiation, followed by bilateral oblique cone down boost using 10-MV X rays with a daily fraction of 1.8–2 Gy to a total dose of 50–70 Gy. Gross tumor volume included the primary tumor and metastatic regional lymph nodes, and clinical target volume included the elective nodal area: supraclavicular and upper mediastinal regional lymph node stations for cervical or upper thoracic tumors; supraclavicular, upper and lower mediastinal regional, and paracardial lymph node stations for middle thoracic tumors; and upper and lower mediastinal regional, paracardial, left gastric and celiac lymph node stations for lower thoracic tumors.

### Assessments

Acute toxicity, including complete blood cell count and serum chemistry profile, was assessed on a weekly basis during dCRT. Toxicities were recorded according to the Common Terminology Criteria for Adverse Events v4.0. Late toxicities were recorded using the Radiation Therapy Oncology Group (RTOG)/European Organisation for Research and Treatment of Cancer (EORTC) late radiation morbidity scoring scheme. Late toxicity was defined as occurring >90 days after treatment initiation.

The following evaluations were performed until disease progression was observed every 3 months for the first year and every 6 months thereafter: physical examination, toxicity assessment, complete blood cell count, serum chemistry profile, endoscopy of the esophagus and CT of the neck, chest and abdomen.

### T-Factor and resectability

Before performing a survival analysis, the T-factor was rediagnosed by an experienced radiologist using the pretreatment CT scans. The clinical decision during the actual treatment regarding resectability, operability and treatment strategy was made by surgeons after discussion at the multidisciplinary tumor conference and used in this analysis. The cases were classified into three groups according to the following definitions: resectable, meaning that complete resection was expected; borderline-resectable, meaning that tumor invasion to surrounding normal tissue is possible, and there is concern about the ability to perform a complete resection; and unresectable, meaning that tumor invasion to surrounding normal tissue is definite, and incomplete resection is expected.

### Statistical analysis

Survival analysis was performed using the Kaplan–Meier method, and the time to event was calculated from the start of the treatment. Differences in survival times between groups were examined using the log-rank test. A *P* value < 0.05 was regarded as a significant difference. All statistical analyses were performed with Easy R (EZR; Saitama Medical Center, Jichi Medical University, Saitama, Japan), which is a graphical user interface for R (The R Foundation for Statistical Computing, Vienna, Austria). More precisely, EZR is a modified version of R commander designed to add statistical functions frequently used in biostatistics [14]. To compare overall survival among resectable, borderline-resectable and unresectable patients, an intent-to-treat analysis was performed, excluding patients who were supposed to receive induction CRT but instead received dCRT due to a poor response.

## RESULTS

### Patient characteristics

A total of 104 patients were identified from our database and included in this analysis. There were 88 males and 16 females. The median age was 68 years (range 48–84). Tumor locations were cervical/cervical and upper thoracic esophagus 3/1, upper thoracic/upper and middle thoracic esophagus 10/12, middle thoracic/middle and lower thoracic esophagus 52/14, and lower thoracic esophagus 12. Thirty-four patients were classified as resectable, 35 were borderline-resectable and 35 were unresectable. The patient characteristics are listed in Table 1.

**Table 1:** Patient characteristics according to resectability

	**Resectable**	**Borderline-resectable**	**Unresectable**
	(*n* = 34)	(*n* = 35)	(*n* = 35)
Age			
Median/range (years)	70/56–84	67/48–84	67/51–82
Sex			
Male/female	28/6	29/6	31/4
Performance status			
0/1/2/3	9/16/8/1	8/25/2/0	7/21/5/2
T-Factor^a^			
1/2/3/4	14/11/9/0	0/2/18/15	3/3/1/28
N-Factor			
0/1/2/3	21/6/6/1	6/11/12/6	2/11/9/13
Stage			
1/2/3/4	18/6/10/0	0/1/28/6	0/0/19/16
Operability^b^			
Operable/inoperable	22/12	33/2	NA^e^
Chemotherapy			
PF-q4w/PF-low dose^c^	22/12	11/24^d^	10/25

^a^Two T2 patients in borderline-resectable had large lymph node metasitasis with probable invasion to surrounding tissues and 7 T1–3 patients in unresectable had cervival or supraclavicular lymph node metastasis; ^b^Operability was judged by surgeons according to patient’s medical condition including comorbidity. This does not mean resectability; ^c^PF-q4w, 2 cycles of cisplatin and 5-fluorouracil every 4 weeks; PF-low dose, cisplatin and 5-fluorouracil 5 days a week for 20 days; ^d^Fifteen patients received definitive chemoradiotherapy due to poor response after induction chemoradiotherapy; ^e^NA, not available/operability was not fully evaluated in unresectable patients.

### Response, toxicity and survival

Complete response (CR) was achieved in 44 patients (42%; 95% confidence interval 33–52%) 1–2 months after the completion of dCRT. Grade 3 or greater acute oral mucositis, esophagitis and hematological toxicities were observed in 12, 17 and 25 patients (resectable, 4, 7 and 5; borderline-resectable, 4, 3 and 10; unresectable, 4, 7 and 10), respectively. Grade 2 or greater late toxicities, such as pericardial effusion, pleural effusion and radiation pneumonitis, were observed in 9, 8 and 7 patients (resectable, 2, 4 and 3; borderline-resectable, 4, 4 and 3; unresectable, 3, 0 and 1), respectively. In total, 18 patients (17%) suffered Grade 2 or greater cardiopulmonary late toxicity and 7 patients (7%) suffered Grade 3 cardiopulmonary late toxicity (resectable, 6 and 1; borderline-resectable, 8 and 4; unresectable, 4 and 2, respectively).

**Fig. 1.**
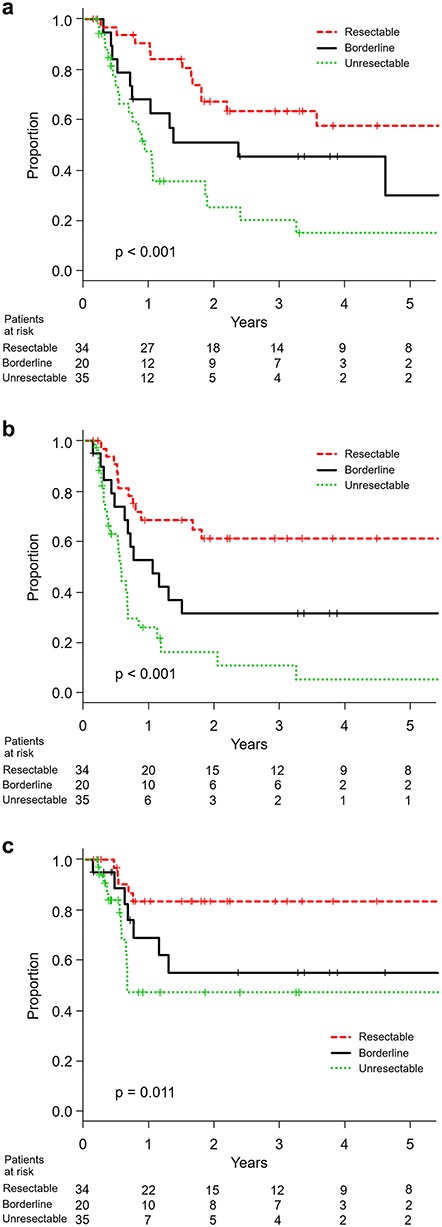
(**a**) Overall survival, (**b**) progression-free survival and (**c**) locoregional control in resectable, borderline-resectable and unresectable patients.

**Fig. 2.**
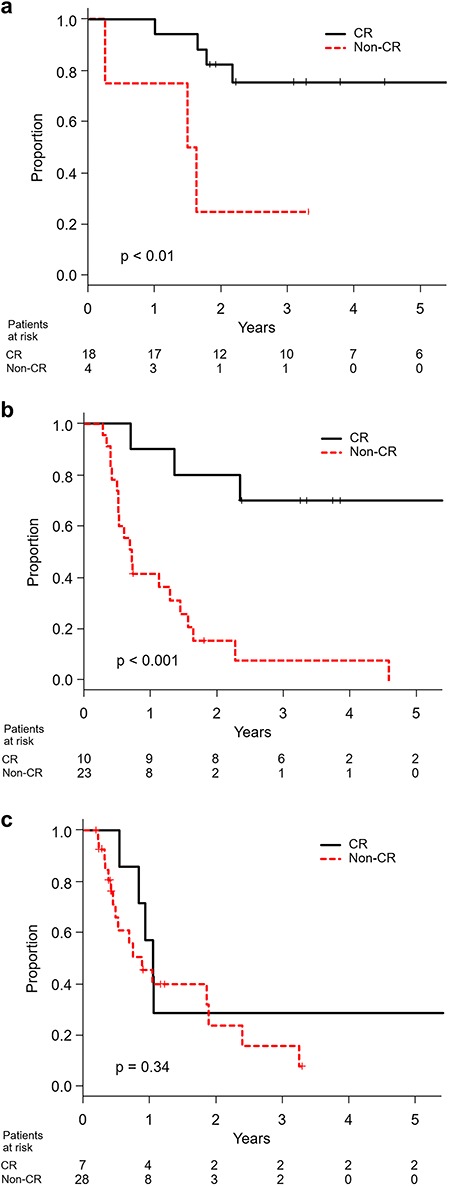
Overall survival in CR and non-CR patients with (**a**) resectable, (**b**) borderline-resectable and (**c**) unresectable disease.

At the time of this analysis, 59 patients were dead and 45 were censored including 14 lost to follow-up within 12 months after the start of treatment. With a median follow-up period of 28 months in survivors, the median survival time for all patients was 13 months. The 3-year overall survival proportions in resectable, borderline-resectable and unresectable patients were 64% (95% confidence interval 44–78%), 46% (95% confidence interval 23–66%) and 21% (95% confidence interval 7–39%), respectively (*P* < 0.001). The progression-free survival and local control curves for resectable, borderline-resectable and unresectable patients were separated accordingly (Fig. 1). With a median follow-up period of 45 months in CR patients, the overall survival for operable borderline-resectable patients with CR and non-CR was significantly different (*P* < 0.001), with 3-year survival proportions of 70% (95% confidence interval 33–89%) and 8% (95% confidence interval 1–27%), respectively. Those for operable resectable patients were 76% (95% confidence interval 47–90%) and 25% (95% confidence interval 1–67%), respectively (*P* < 0.01). However, the overall survival for unresectable patients with CR and non-CR was not different (*P* = 0.34), with median survival times of 12 months and 10 months, respectively (Fig. 2).

## DISCUSSION

One of the major issues in the treatment of esophageal cancer is lack of accurate and established decision criteria for resectability [11–12]. This results in large inter-observer variations among diagnostic radiologists and surgeons, and leads to uncertainty in the decision of resectability. This is the reason why the concept of borderline-resectable disease was proposed with a treatment strategy being sought that is different from resectable and unresectable disease. Yokota *et al*. [13] reported the concordance of clinical diagnosis of T4 for 48 cases with locally advanced esophageal cancer entered in a phase 2 study. Ninety percent of cases were diagnosed as clinical T4 by attending physicians, while 33–75% (median 72%) of cases were diagnosed as T4 by six external reviewers. Discordant diagnoses between attending physicians and reviewers occurred in 33% of all cases, and these cases should be considered borderline-resectable. This discordance may also be partly explained by a deviation from one of the general rules of the UICC-TNM staging system. In general, CT diagnosis of clinical T4 includes both probable and definite invasion to surrounding normal tissues, and T3 includes possible but indefinite invasion to surrounding normal tissues, contrary to the rule of the UICC-TNM staging system: ‘If there is doubt concerning the correct T, N, or M category to which a particular case should be allotted, then the lower (i.e., less advanced) category should be chosen.’ Thus, the borderline-resectable group included both T3 and T4 patients. As shown in Table 1, 35 out of 104 patients were determined to be borderline-resectable. These 35 patients were initially diagnosed as T3 in 18, and T4 in 15 patients showing substantial staging migration. The 2 patients diagnosed as T2 were exceptional and had large lymph node metastasis with probable invasion to surrounding tissues.

The 3-year overall survival proportions in patients with borderline-resectable and unresectable disease were different (46 and 21%, respectively). Hironaka *et al*. reported the results of a nonrandomized comparison between dCRT and radical surgery in patients with T(2–3)N(0–1)M0 disease and found a 3-year overall survival proportion of 49% in the dCRT group [15]. Kato *et al*. reported that the 3-year overall survival proportion was 45% in a phase II study of stage II–III cases [16]. These results suggest that the overall survival for borderline-resectable cases is similar to that for stage II–III (excluding T4) cases and should be treated the same as stage II–III cases when treated by dCRT.

Additionally, induction chemotherapy with or without concurrent CRT has been investigated for borderline-resectable and unresectable cases to perform complete resection for responders. Stahl *et al*. reported the results of a randomized study comparing induction chemotherapy followed by dCRT vs induction chemotherapy followed by concurrent CRT and surgical resection in T(3–4)N(0–1)M0 esophageal cancer. The overall survival was equivalent between the two groups, although the local progression-free survival was better in the surgery group, with significantly higher treatment-related mortality in the surgery group than in the dCRT group (13 vs 3.5%, *P* = 0.03) [17]. Yokota *et al*. reported the updated results of a phase 2 study of chemoselection with docetaxel plus 5-FU and cisplatin (DCF) and subsequent conversion surgery for borderline-resectable and unresectable disease [10], following the encouraging results of a retrospective analysis of induction DCF followed by surgery for borderline-resectable disease [8]. The 3-year overall survival proportion was 47% for all cases, including both induction DCF followed by dCRT and induction DCF followed by surgery. The 3-year overall survival proportion for responders who underwent complete resection was 71%, while that for those who did not undergo complete resection was 30%. Interestingly, the 3-year overall survival proportions of CR and non-CR for borderline-resectable cases in our study were 70 and 8%, respectively. The observed 3-year overall survival proportion of 70% was quite encouraging and was comparable to the 3-year overall survival proportion of 62% for stage II–III cases treated by induction chemotherapy followed by surgery, which is a standard treatment for resectable disease in Japan [18]. Due to the limited number of patients, selection biases and other limitations inherent to a single arm study and a retrospective analysis, a firm conclusion could not be drawn. However, until now, it has yet to be determined whether overall survival after induction chemotherapy followed by surgery for responders is superior to that after dCRT in CR cases. A randomized phase 3 study comparing induction DCF followed by dCRT or induction DCF followed by surgery vs dCRT with cisplatin and 5-FU is underway (Japan Clinical Oncology Group (JCOG) 1510, UMIN000031165). This study will clarify the role of induction DCF, but it remains an open question whether surgery would be necessary for CR cases after dCRT.

In conclusion, the overall survival in CR patients with borderline-resectable disease after dCRT was encouraging. Further investigation is warranted to accurately estimate the efficacy of dCRT for borderline-resectable cases and to find a patient subgroup that benefits from esophagus-preserving treatment.
